# Village-Indigenous Chicken Bacterial Carriage after the Heavy Rains of 2018, Kenya: Indicator on Environmental Contamination with Pathogenic/Zoonotic Bacteria

**DOI:** 10.1155/2022/5437171

**Published:** 2022-07-09

**Authors:** Acsa Igizeneza, Lilly Caroline Bebora, Philip Njeru Nyaga, Lucy Wanjiru Njagi

**Affiliations:** College of Agriculture and Veterinary Sciences, Faculty of Veterinary Medicine, Department of Veterinary Pathology, Microbiology and Parasitology, University of Nairobi, P.O. Box: 29053-00625, Nairobi, Kenya

## Abstract

Food borne diseases are one of the major human disease conditions worldwide. Most of them are of bacterial origin and chickens are a major source of such bacteria; they are consumed at high rate worldwide and tend to harbor the zoonotic bacteria without showing signs of illness. Running rain water tends to increase environmental contamination, since it carries various substances from one area to another; this results in village-indigenous chickens picking more bacteria from the environment as they roam/scavenge around for food. Thus, after the rain, the chickens' intestinal contents may contain more bacteria quantity-wise and type-wise. This study was carried-out to determine whether that was the case after heavy rains of 2018.120 intestine samples were collected from indigenous chickens from three slaughterhouses in Nairobi for bacterial quantification using the Miles and Misra technique; bacterial isolation and identification were carried out using standard bacteriological procedures. Intestines from the slaughterhouses had different mean bacterial counts: Kangemi had the highest (1.3 × 10^12^ colony-forming units per ml), followed by Burma (5.6 × 10^11^), then Kariokor (4.7 × 10^11^). *E*. *coli* was the most isolated at 85.8%, followed by genera *Staphylococcus* (55%), *Streptococcus* (43.3%), *Bacillus* (41.66%), *Listeria* (38.3%), *Proteus* (24.16%), *Klebsiella* (7.5%), *Campylobacter* (2.5%), *Pseudomonas* (6%), and *Streptobacillus* (0.83%). The study showed that the indigenous chickens carry a variety of bacteria in types and numbers, some of them being zoonotic. Apart from picking more bacteria as a result of environmental contamination during rainy season, the weather and bird-handling further stress the birds, thus contributing to higher bacterial multiplication and higher bacterial carriage. If slaughter is not done right, these intestinal bacteria can easily cause contamination of respective chicken meat; thus, if pathogenic, it can cause food poisoning to consumers of the meat. Therefore, it is recommended that precaution be taken while slaughtering chickens for consumption. In addition, where possible, free-range indigenous chickens be confined during rainy seasons to reduce their exposure to contaminated environment.

## 1. Background

Poultry population in Kenya contributes 30% of agricultural GDP (where 26% of overall GDP is from agriculture). Nairobi city is known to be the major destination of most poultry, particularly of indigenous types from other counties [1]. These chickens are normally kept under free-range system of management in villages [2, 3] and serve as a source of protein to humans in the form of meat and eggs [4]. They also have other diverse functions in the community [3, 5]. The traditional free-range system is the least capital intensive system requiring minimal financial input, hence affordable to even the resource-poor persons [2, 3]; they scavenge for their own feed with little or no supplementation.

Just like other animals and humans, chickens carry bacteria in their guts [6, 7], reproductive systems [8, 9], and respiratory tracts [10, 11], mostly as normal flora. These bacteria, which are nonpathogenic to the chickens, coexist and play an important role to their hosts, chickens [12–14].

Although they tend to occur as commensals in indigenous chickens, some of the bacteria, for example: *Escherichia coli*, *Campylobacter* spp*, Listeria* spp, and *Salmonella* serotypes, are of public health importance—they can cause disease in humans, depending on their pathogenicity and concentration [15–18].

These indigenous birds contribute towards human diseases and bacterial contamination of environment in various ways. During evisceration of such birds, at slaughter, the zoonotic bacteria may contaminate the slaughterhouse environment and utensils, resulting in meat contamination [18–20] and cause food poisoning to humans who consume the contaminated meat. Also, since they roam about the village, sometimes long distances, defecating everywhere, free-range indigenous chickens are normally a source of contamination to a wider environment, resulting in spread of bacteria, including disease-causing ones [3, 6]. The situation is worsened if these bacteria are resistant to antibiotic(s) and/or disinfectant(s) as it will be difficult to treat the resultant disease(s) [20–24].

There is high probability that bacterial load and type in the environment would increase during wet weather, particularly during heavy rains, as a results of rain water flowing from highlands to lowlands, contributing to the spread of different materials and wastes, insects, a wide variety of herbs, among others [25–27], which can be harboring different types of bacteria among other substances [28]. There is, therefore, high possibility of chickens picking them up as they scavenge.

The possible occurrence of parasitism, both endo- and ecto-, acquired mainly during the wet season, can lead to discomfort and cause the birds not to eat well. This will worsen the immunity of already immune-suppressed birds, thus resulting in increased bacterial load [29–31]. This will give an opportunity for some of the bacteria to cause diseases to the host and also allow other pathogenic bacteria to establish themselves [14].

In year 2018, between March and May, there were excessive rains in Kenya (countrywide) which resulted in flooding and mudslides, more than what was experienced in 1961, 1984, 2006 and 1984 *El Niño* [25, 32], the heavy rains were also recorded in the neighboring countries (Uganda, Burundi, Rwanda, Ethiopia, and Somalia) [33]. Although a number of researchers have isolated bacteria from chicken in Kenya [7, 9, 10], none of them focused on doing it after heavy rains. Thus, this study was found necessary in order to establish and document such data. It covered both bacterial types and counts from chickens.

## 2. Materials and Methods

### 2.1. Sample Collection

The study was cross sectional; 120 intestines of slaughtered village - indigenous chickens were obtained from three slaughterhouses: Kariokor, Burma, and Kangemi, in Nairobi. They were transported aseptically in cool boxes to the departments of veterinary, pathology, microbiology, and parasitology, where they were processed for bacterial counting and identification. While collecting the samples from the slaughterhouses, details on origins/sources of the slaughtered birds, their stay at the slaughterhouse before being bought, and whether or not any feed supplementation given were documented.

### 2.2. Total Bacterial Counting

Total bacterial counting (cfu/ml) was done to enumerate the bacterial load using the method of Miles and Misra [34] as follows: one (1) gram of intestinal contents was placed in 9 ml of normal saline (0.85% sodium chloride). The suspension was mixed thoroughly by vortexing; then ten-fold serial dilutions were made (from 10^−1^ to 10^−10^) in test tubes.

Then, using a micropipette which drops 25 microlitres (equivalent to 40 drops to an ml, i.e., a drop represents 1/40^th^ of a ml), two drops from each dilution were dropped separately onto nutrient agar (in petri dish), which was divided into four quadrants. The plates were then incubated at 37°C overnight, after which colony counting was done at drops which had countable isolated colonies; counts of the duplicate drops were averaged and quantification of bacteria in the original suspension was made.

### 2.3. Bacterial Isolation and Identification

Bacterial isolation was done by using different growth media: general medium used was blood agar, and selective and/or differential media were mannitol salt agar, MacConkey, salmonella-shigella agar, cystine tellurite blood agar, sodium azide crystal violet blood agar, thiosulphate citrate bile salts sucrose Agar, Camp Karmali, and eosin methylene blue. To screen *E*. *coli* O157 : H7, MacConkey sorbitol agar was used; the suspected colonies were typed using respective sera Prolex™ *E*. *coli* 0157. Selenite broth for *Salmonella* serotypes and alkaline peptone water for *Vibrio* spp were used as enrichment media [15]. To increase chances of isolating *Listeria* organisms, samples were subjected to cold enrichment at 4°C overnight [15, 35, 36]. Different biochemical tests were then used to identify the isolated bacteria including oxidase, catalase, indole, methyl red, citrate, and urease; reaction on triple sugar iron agar; reaction on sulphur indole motility medium; and CAMP test was used for *Listeria monocytogenes* and *Streptococcus* spp isolates, and hanging drop motility test for *Listeria* spp as given by Bergey's manual for systemic bacteriology, Holt and Williams [35], and Cowan and Steel's manual [36].

### 2.4. Statistical Analysis

Descriptive statistics was used to analyze obtained data. Bacterial counts were analyzed by one way analysis of variance (ANOVA) to compare the arithmetic means of bacterial counts. Bacterial isolation findings were analyzed by chi square test using statistical package for social sciences (SPSS statistical program), to check the association of the isolates from three different slaughter houses.

## 3. Results

### 3.1. Holding of Chickens at the Market and Feed Supplementation

At Kariokor slaughterhouse, chickens were normally sold the same day they arrive, however, in Burma and Kangemi slaughterhouses, chickens were staying in the slaughterhouse for few days before sale. During this time, they were being fed on grains mostly maize and water, no additional foods nor supplements were given to them.

### 3.2. Total Bacterial Count

**Figure 1:**
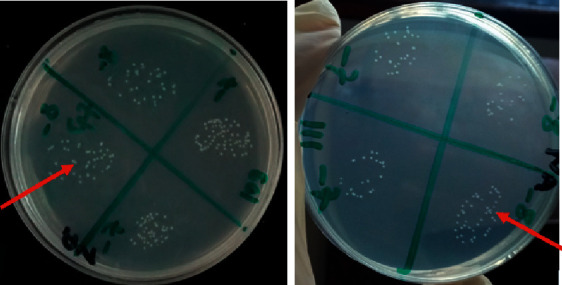
Countable colonies on nutrient agar media as pointed by the red arrows.

Bacterial counting from the three slaughterhouses ranged from 10^4^ to 10^12^ colony-forming units per milliliter (cfu/ml); the birds had different intestinal bacterial concentrations. Chickens from Kariokor slaughterhouse had lower bacterial carriage than those from other slaughterhouses as shown in Table 1.  Figure 1 shows countable colonies from different dilutions produced on nutrient agar plates after overnight incubation.

Table 1 shows the number of chickens showing the approximate concentration (cfu/ml) per slaughterhouse. Respective means for the study of chickens in the three slaughterhouses were significantly different (*p*=0.0266). Results of homogeneity test after analyzing arithmetic means of the counts, using analysis of variance (ANOVA), showed that the counts from Kariokor (4.7 × 10^11^) and Burma (5.6 × 10^11^) slaughterhouses were not statistically different but different from the counts from Kangemi slaughterhouse (1.3 × 10^12^).

### 3.3. Prevalence of Bacterial Isolates

**Figure 2:**
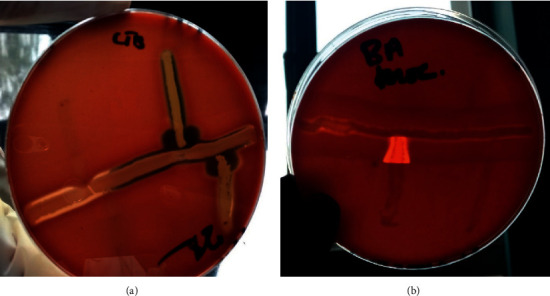
CAMP test results of the isolates against *Staph*. *aureus* where: (a) is the shovel shape of *Listeria monocytogenes* and (b) is the arrow shape of *Strep*. *agalactiae.*

**Figure 3:**
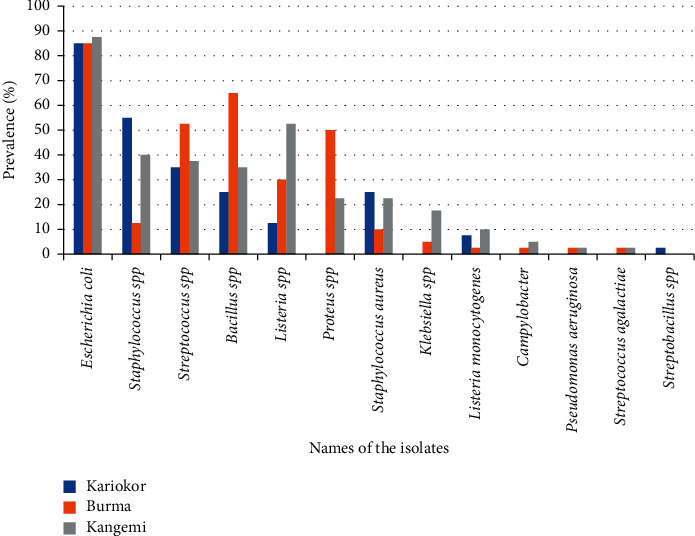
Prevalence rates of isolates per slaughterhouse.

From the 120 intestinal samples collected (40 per slaughterhouse), thirteen genera were identified among others.  Figure 2 shows the camp test results for some isolates.  Figure 3 gives prevalence rates of the isolates per slaughterhouse, while Table 2 gives prevalence of bacteria isolated from the slaughterhouses and their respective chi square values. Overall, *E*. *coli* was the highest isolated at 85.8%, followed by both *Bacillus* spp and *Streptococcus* spp other than *Strept*. *agalactiae* at 41.7% each, *Staphylococcus* spp other than *Staph*. *aureus* at 34.2%, *Proteus* spp at 24.2%, *Listeria* spp other than *L*. *monocytogenes* at 31.7%, *Staph*. *aureus* at 17.5%, *Klebsiella* spp at 7.5%, *Listeria monocytogenes* at 6.7%, *Campylobacter* spp at 2.5%, *Streptococcus agalactiae* at 1.7%, *Pseudomonas aeruginosa* at 6%, and lastly *Streptobacillus* spp at 0.8%.

Bacteria isolated from Kariokor slaughterhouse were as follows: the most prevalent was *Escherichia coli* (34/40; 85%), followed by *Staphylococcus* spp other than *Staph*. *aureus* at 55% (22/40) and *Streptococcus* spp at 40% (16/40). The most isolated bacteria from Burma slaughterhouse were as follows: *E*. *coli* (34/40; 85%), followed by *Bacillus* spp at 65% (26/40), *Streptococcus* spp at 52.5% (21/40), and *Proteus* spp at 50% (20/40). Bacteria isolated from Kangemi slaughterhouse, the most prevalence were as follows: *E*. *coli* (35/40; 87.5%), followed by *Listeria* spp at 52.5% (21/40), and *Staphylococcus* spp at 40% (16/40). More details are indicated in Table 2 and Figure 2.

Efforts were made to isolate *Vibrio* spp (TCBS media), *Salmonella* serotypes, and *Shigella* spp (SSA media), but the bacteria were not isolated. Some *E*. *coli* isolates produced pale colonies on Sorbitol MacConkey but, on typing with respective antiserum, they were not serotype O157 : H7. Isolates that were further confirmed are the only ones reported in this study.

## 4. Discussion

Bacterial carriage of the test chicken intestines ranged between 10^4^ and 10^12^ colony forming units per millimeter (cfu/ml). The results have shown no difference in bacterial counts between intestines obtained from Kariokor and Burma slaughterhouses; while those from Kangemi had higher counts, as demonstrated by ANOVA test. Mean counts from Kariokor and Kangemi were 4.7 × 10^11^ and 5.65 × 10^11^ cfu/ml, respectively; while the one from Kangemi was 1.32 × 10^12^ cfu/ml. There are two possible reasons for this: (1) it may be that the indigenous chickens from Kangemi were exposed to higher number of bacteria before being transported to the market; meaning that they came in already carrying heavy loads of the respective bacterium/bacteria, having scavenged from heavily-contaminated ground [28] or (2) the birds could have acquired more bacteria at the slaughterhouse as a result of poor holding conditions during their stay before being sold; thus, there could have been cross-infections among them, through defecation or oropharyngeal excretions which concurs with the findings of Kim et al. [27]. Although this could also happen during dry season and transportation from the source, the possibility that the chickens' bacterial carriage was higher due to increased environmental contamination and stress, after the rainy season, cannot be ruled out, leading to the kept birds excreting more bacteria, thus, resulting in enhanced cross-infection between them. Not to forget the stress developed due to the time spent in the slaughterhouse and transport time, which could cause the decrease in immunity. Information gathered at the time of sample collection showed that, for the three slaughterhouses, the chickens were sourced from different geographical locations.

A study done by Proietti et al. [37] has demonstrated bacterial counts from chicken intestines of *n* × 10^6^ cfu/ml. That study showed a lower count compared to the one obtained in the current study which had means of *n* × 10^11^ and *n* × 10^12^. As mentioned in literature review, the heavy rains of 2018, between March-May, in Kenya, caused destruction of toilets and over flooding of rivers; this could have contributed to the spreading of different materials that carried bacteria from one location to another [28, 31–33]. The indigenous chickens would then be infected as they fed from the highly contaminated environment. The study done by Singh et al. [38] showed that there is an increase in bacterial prevalence in poultry fecal samples collected in rainy seasons compared to winter and summer seasons. Also, there is high probability that the indigenous chickens were having reduced immunity as the result of climate change (due to wetness and cold experienced during and after the rainy season), which resulted in them becoming vulnerable to bacterial establishment and multiplication [30, 31]; hence, increase in the bacterial carriage.

The findings from the current investigation were not different from the one got by Smith and Crabb [39] even though it was not mentioned whether that study was conducted during rainy season or not. They documented a total bacterial count between 10^3^ and 10^10^ cfu/ml in chicken feces. Apart from this, there is minimal literature on intestinal bacterial counts and all of them are from other countries; this is the first study done on total intestinal bacterial counts from indigenous chicken in Kenya.

Among the identified bacterial isolates, *E*. *coli* was the most prevalent at 85.8%, followed by *Staphylococcus* spp at 55%; *Streptococcus* spp at 43.3%, *Bacillus* spp at 41.7%, *Listeria* spp at 38.3%, *Proteus* spp at 24.2%, *Klebsiella* spp at 7.5%, *Campylobacter* spp at 2.5%, *Pseudomonas aeruginosa* at 1.7%, and lastly *Streptobacillus* spp at 0.8%.

Being a normal habitant of human and animal gastrointestinal tract [15], having *E*. *coli* as the most isolated organism at 85.5% is not surprising because fecal material normally has high loads of *E*. *coli*. This is supported by Furtula et al., [40] who demonstrated presence of high numbers of *E*. *coli* in chicken litter. Since the organisms are always found in the intestinal tract, they are taken as a good microbial indicator of the potential presence of disease caused by bacteria and also show the general sanitary quality of the food since they are closely associated with fecal contamination [15]. However, it is documented that there are pathogenic *Escherichia coli* strains, including *E. coli* O157 : H7, that cause various degrees of diarrhea and septicemia in both animals and humans [15, 18, 20]. There was no difference in isolation rates of *E*. *coli* among the three slaughterhouses (*p*=0.8).

*Streptococcus* spp were also isolated at a fairly high rate (41.7%), with *Streptococcus agalactiae* at 1.66%. Isolation of *Streptococcus* spp in chicken intestines is normal as documented by Devriese et al., [6] who showed presence of *Streptococcus* spp in the intestines of healthy-appearing chicks of ages 3 weeks (30%) and 12 weeks (27%). It is, however, associated with both chronic and acute (septicaemic) disease, causing mortality rates between 0.5% and 50% in poultry [41]. In humans, it is known to cause respiratory tract infections such as acute sinusitis, acute otitis media, pharyngitis, community-acquired pneumonia, and acute bronchitis among others [42]. *Streptococcus agalactiae* can cause postpartum infection and neonatal septicaemia in humans [15]. In this study, there was no significant difference between isolation rates of *Streptococcus* organisms in general (*p*=0.2) and *Strep*. *agalactiae* in particular (*p* value of 0.6) among the three study slaughterhouses.

*Staphylococcus* spp are normal flora of many animals including chickens; the organisms are however, known to be opportunistic and can cause serious diseases under adverse circumstances. *Staphylococcus aureus* is known to cause intoxication in humans after consuming contaminated food [43]. They can also cause skin infections and life-threatening conditions like endocarditis, toxic shock syndrome, and necrotizing pneumonia [44, 45]. In this study, *Staphylococcus* spp were isolated at 55% (*Staph*. *aureus* at 19.2% and other *Staphylococcus* spp at 35.8%). In previous studies *Staph. aureus* was isolated from chicken at 22% [46], which is slightly different from this study's findings. The isolation rate of *Staphylococcus aureus* among the slaughterhouses was not significantly different (*p*=0.2). The organisms were mostly isolated from Kariokor slaughterhouse at 55%.

In this study, *Listeria monocytogenes* was isolated at 6.7%; isolation rates not being different among the three slaughterhouses (*p* value = 0.4). A study carried out by Njagi [24] documented presence of *Listeria* spp in slaughtered indigenous chickens at 12.5%, which is different from what was found in this study. Furthermore, in the current study, other *Listeria* spp were also isolated at 31.7% with significant differences between the slaughterhouses (*p*=0.001). Also, a study done in Germany showed presence of *Listeria* spp in healthy chickens [47]. *Listeria* species' isolation rate at Kariokor slaughterhouse was lower than that of the other two markets; meaning that the chickens sold here were minimally exposed to the organism. *Listeria* organisms have been documented as one of the sources of human food poisoning associated with meningitis and endocarditis [15, 48]; the organisms are normally found in soil, plant materials, and silages—chickens may get infected through feeding on these [15].

*Campylobacter* spp, especially *Campylobacter jejuni* and *Campylobacter coli*, are of public health importance; they can cause food poisoning to the consumers; they are known to cause human gastroenteritis worldwide [49]. Chickens are known to be a major source of campylobacter infection to humans; most of them being asymptomatic carriers; thus, they are a threat to human [7, 15]. A study done by Zhao et al. [50] has documented prevalence of *Campylobacter* spp of 70.7% in chickens from Greater Washington D.C. In this study, *Campylobacter* spp were isolated at low rate of 2.5%, not because the chickens were free from the organism but may be because of the isolation conditions used which did not provide a favorable environment [15].

Other bacteria isolated in this study were: *Pseudomonas aeuroginosa* (1.7%), *Klebsiella* spp (7.5%), *Streptobacillus* spp (0.8%), *Bacillus* spp (41.7%), and *Proteus* (24.6%). It has been recorded in the literature that they can be isolated from chickens [27, 45, 51–53]. They can cause diseases in chickens and humans, if found in large amounts. *Pseudomonas aeuroginosa* can cause corneal ulcers if the eyes got infected by the organism; they also contaminate wounds [54]. Most of *Bacillus* spp are harmless and/or opportunistic pathogens (*B*. *cereus* and *B*. *licheniformis*) which can cause food-borne diarrhea in humans, with exception of some which are very harmful, for example, *B*. *anthracis*. There is evidence that some *Bacillus subtilis* strains are used to control against *Clostridium perfringens* infection in chickens, [55] and *B*. *circulans* has inhibition activity to *Campylobacter jejuni* [56]. *Proteus mirabilis* can cause respiratory tract and wound infections as an opportunistic bacterium. From this study, isolation rates of *Bacillus* spp and *Proteus* spp among the three slaughterhouses was significantly different (*p* ≤ 0.001).

Though the bacterial types isolated and identified in this study were not different compared to what other investigators found, the high numbers/concentrations, carried by the chickens, could have been contributed by the rains; hence, as mentioned above, care needs to be taken in managing chickens in rainy seasons.

## 5. Conclusion

This study, therefore, has shown that indigenous chickens carry different types of bacteria some of which are zoonotic; they can be transmitted to humans either directly from the birds or through ingestion of contaminated chicken meat. It also cautions on the possibility that the heavy bacterial carriage of the tested indigenous chickens could be as a result of environmental contamination due to heavy rains; the chicken getting more infected as a result of scavenging on the contaminated ground. It is, therefore, advisable that, where possible, movement of indigenous village chickens be restricted during rainy seasons, to minimize the environmental exposure. It is also important that policy makers come up with guidelines with respect to management of the village chickens, towards reduction of environmental contamination.

## Figures and Tables

**Table 1:** Number of chickens that had the respective total bacterial concentration.

Slaughterhouse	Total number of samples tested	Number of birds that had respective counts (cfu/ml) plus respective percentage in brackets						
		*n* × 10^4^	*n* × 10^6^	*n* × 10^8^	*n* × 10^9^	*n* × 10^10^	*n* × 10^11^	*n* × 10^12^
Kariokor	38	4 (10.5%)	4 (10.5%)	1 (2.6%)	1 (2.6%)	3 (7.9%)	22 (57.9%)	3 (7.9%)
Burma	36	—	—	3 (8.3%)	1 (2.8%)	19 (52.8%)	11 (30.6%)	2 (5.6%)
Kangemi	40	—	—	1 (2.5%)	12 (30%)	15 (37.5%)	8 (20%)	4 (10%)
Combined data	114	4 (3.5%)	4 (3.5%)	5 (4.4%)	14 (12.3%)	37 (32.5%)	41 (36.0%)	9 (7.9%)

*n* is the unit figure that needs to be multiplied by the respective power 10; cfu/ml is colony forming units per millimeter.

**Table 2:** Prevalence of bacteria isolated from Kariokor, Burma, and Kangemi slaughterhouses and their chi square values.

Bacteria isolated	Number of the isolates (% of total); *n* = 120 (%)	Kariokor (% of respective total); *n* = 40 (%)	Burma (% of respective total); *n* = 40 (%)	Kangemi (% of respective total); *n* = 40 (%)	*p* values	*χ* ^2^
*Escherichia coli*	103 (85.8)	34 (85)	34 (85)	35 (87.5)	0.8	0.46^NS^
*Proteus* spp	29 (24.2)	0 (0)	20 (50)	9 (22.5)	≤0.001	23.23^*∗∗∗*^
*Staphylococcus aureus*	23 (19.2)	10 (25)	4 (10)	9 (22.5)	0.19	3.34^NS^
Other *Staphylococcus* spp	43 (35.8)	22 (55)	5(12.5)	16 (40)	≤0.001	23.42^*∗∗∗*^
*Streptococcus agalactiae*	2 (1.7)	0 (0)	1 (2.5)	1 (2.5)	0.60	1.03^NS^
Other *Streptococcus* spp	50 (41.7)	14 (35)	21 (52.5)	15 (37.5)	0.17	4.05^NS^
*Listeria monocytogenes*	8 (6.7)	3 (7.5)	1 (2.5)	4 (10)	0.40	1.88^NS^
Other *Listeria* spp	38 (31.7)	5 (12.5)	12 (30)	21 (52.5)	0.001	14.7^*∗∗∗*^
*Pseudomonas aeruginosa*	2 (1.66)	0 (0)	1 (2.5)	1 (2.5)	0.60	1.02^NS^
*Streptobacillus* spp	1 (0.83)	1 (2.5)	0 (0)	0 (0)	0.37	2^NS^
*Bacillus* spp	50 (41.66)	10 (25)	26 (65)	14 (35)	≤0.001	19.38^*∗∗∗*^
*Klebsiella* spp	9 (7.5)	0 (0)	2 (5)	7 (17.5)	0.009	9.37^*∗∗∗*^
*Campylobacter* spp	3 (2.5)	0 (0)	1 (2.5)	2 (5)	0.36	2.051^NS^

NS means no significant difference of isolation rates between the markets; ^*∗∗∗*^means that there is significant difference, with respect to isolation rates, among the markets, at *p* value of 0.05.

## Data Availability

Data can be available upon request from the corresponding author.
